# Antibacterial activities of bacteriocins: application in foods and pharmaceuticals

**DOI:** 10.3389/fmicb.2014.00241

**Published:** 2014-05-26

**Authors:** Shih-Chun Yang, Chih-Hung Lin, Calvin T. Sung, Jia-You Fang

**Affiliations:** ^1^Research Center for Industry of Human Ecology, Chang Gung University of Science and TechnologyTaoyuan, Taiwan; ^2^Pharmaceutics Laboratory, Graduate Institute of Natural Products, Chang Gung UniversityTaoyuan, Taiwan; ^3^Center for General Education, Chang Gung University of Science and TechnologyTaoyuan, Taiwan; ^4^Chronic Diseases and Health Promotion Research Center, Chang Gung University of Science and TechnologyTaoyuan, Taiwan; ^5^Department of Microbiology, Immunology, and Molecular Genetics, University of California, Los AngelesLos Angeles, CA, USA; ^6^Chinese Herbal Medicine Research Team, Healthy Aging Research Center, Chang Gung UniversityTaoyuan, Taiwan

**Keywords:** bacteriocin, protein, natural product, food, cancer treatment

## Abstract

Bacteriocins are a kind of ribosomal synthesized antimicrobial peptides produced by bacteria, which can kill or inhibit bacterial strains closely-related or non-related to produced bacteria, but will not harm the bacteria themselves by specific immunity proteins. Bacteriocins become one of the weapons against microorganisms due to the specific characteristics of large diversity of structure and function, natural resource, and being stable to heat. Many recent studies have purified and identified bacteriocins for application in food technology, which aims to extend food preservation time, treat pathogen disease and cancer therapy, and maintain human health. Therefore, bacteriocins may become a potential drug candidate for replacing antibiotics in order to treat multiple drugs resistance pathogens in the future. This review article summarizes different types of bacteriocins from bacteria. The latter half of this review focuses on the potential applications in food science and pharmaceutical industry.

## Introduction

**Figure 1:**
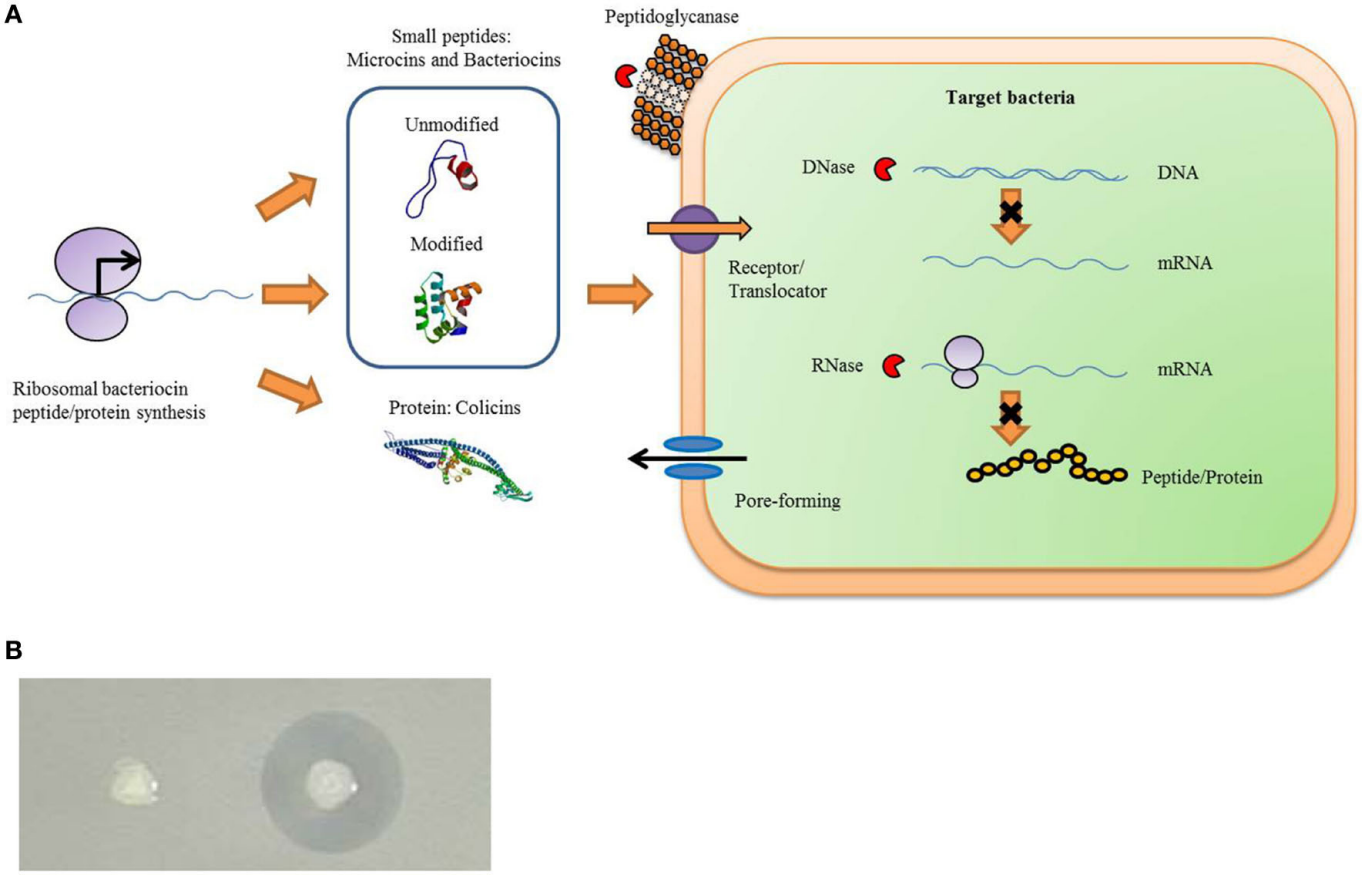
**Bacteriocins function as a natural bacterial immune weapon system**. Gram-positive and Gram-negative can produce many kinds of bacteriocins that allow bacteriocin-producing bacteria to have the ability to inhibit the growth of sensitive bacteria. **(A)** General process of bacteriocins production and antibacterial functions. Bacteriocins are proteins or peptides synthesized by the ribosomal. When released by bacteriocin-producing bacteria, it can become combined with the corresponding receptor on the surface of the sensitive bacteria to kill the bacteria. The sensitive bactericidal mechanisms include the pore-forming type, a nuclease type with DNase and RNase function, and peptidoglycanase type etc. The structure of an unmodified bacteriocin peptide of small molecular weight is subtilisin A (PDB cord 1PXQ). The structure of the modified bacteriocin peptide of small molecular weight is bacteriocin AS-48 (PDB cord 1O83), and the colicin protein structure is colicin Ia (PDB cord 1CII). These structural diagrams are taken from the website of a protein data bank (PDB). **(B)** When the bacteriocin-producing strains are growing on sensitive bacteria LB soft agar, an inhibition zone will be produced around the bacteriocin-producing strains (right colony). However, there is no inhibition zone around bacteriocin non-producing strains (left colony).

There are many antibacterial substances produced by animals, plants, insects, and bacteria, such as hydrogen peroxide, fatty acids, organic acids, ethanol, antibiotics, and bacteriocins. Antimicrobial peptides (AMPs) or proteins produced by bacteria are categorized as bacteriocins. Scant nutrients in the environment trigger microbial production of a variety of bacteriocins for competition of space and resources. Bacteriocins are abundant, have large diversity, and the genes encode ribosomally synthesized antimicrobial peptides or proteins, which kill other related (narrow spectrum) or non-related (broad spectrum) microbiotas as one of the inherent defense system weapons of bacteria (Figure 1) (Cotter et al., 2005). More than 99% of bacteria can produce at least one bacteriocin, most of which are not identified (Riley and Wertz, 2002). The killing ability of bacteriocins is considered a successful strategy for maintaining population and reducing the numbers of competitors to obtain more nutrients and living space in environments. Unlike most antibiotics, which are secondary metabolites, bacteriocins are ribosomally synthesized and sensitive to proteases while generally harmless to the human body and surrounding environment.

Modern society is more conscious of the importance of food safety, as many of the chemical additives used in food may elicit toxic concern; therefore, it is beneficial to claim natural resources and health benefits of diets. The health benefits of natural foods without chemical additives have become more popular; however, most commercially available preservatives and antibiotics are produced by chemical synthesis, and long-term consumption of such products can affect human health as they reduce the counts of bacteria in the gut. Moreover, the use of antibiotics or residues in food is illegal. Unlike chemical preservatives and antibiotics, “generally recognized as safe” (GRAS) bacteriocins, such as nisin, promise safe use as a food preservative in vegetables, dairy, cheese, meats, and other food products, as they inhibit microorganisms contamination during the production process (Deegan et al., 2006; Settanni and Corsetti, 2008).

This review focuses on the classification of bacteriocins from Gram-negative and Gram-positive bacteria. The application of bacteriocin-producing bacteria and bacteriocins from natural resources for human life is also elucidated upon in the report.

## Classification of bacteriocins

### Bacteriocins from gram-negative bacteria

#### Colicins

Colicins are antibacterial proteins produced by bacteria, which can kill bacterial strains closely related to a produced species, in order to reduce environmental competitors for acquiring nutrients and living space. Colicins are organized in three specific domains, an amino-terminal translocation (T) domain, which is implicated in the transfer across the outer membrane via the translocator protein; a central receptor-binding (R) domain, which is bound with a bacterial outer membrane receptor; and a carboxy-terminal cytotoxic (C) domain, which has antibacterial activity (Cascales et al., 2007; Kleanthous, 2010). In order to avoid poisoning by self-produced colicins, specificity immunity proteins will be simultaneously produced to inactivate colicins (Kleanthous, 2010). When a bacterial outer membrane surface has the colicins recognition receptors protein and the translocators protein system, the colicins are transported into the bacteria, which kills it, and are known as sensitive strains. For a particular colicin, non-receptor protein bacteria are classified as resistant strains. Bacteria with a deficiency of translocator protein system are classified as tolerant strains, which produce immunity proteins are classified as immune strains. Resistant, tolerant, and immune strains of bacteria would not be killed by corresponding colicins. There are many colicins found in succession, most of which are encoded on plasmids, while few are located in chromosomes. A typical colicin gene cluster encodes the toxin protein, immunity protein, and lysis gene (Guasch et al., 1995; Kleanthous, 2010). The lysis protein, known as bacteriocin release protein (BRP), can induce the release of colicins from bacteria.

According to translocation across the outer membrane (translocator) system, colicins are categorized into two groups: groups A and B (Table 1). Group A colicins use the Tol protein system (Tol system) to penetrate the outer membrane of sensitive bacteria, for example: colicins E1 to E9, colicin A, K, N… Group B barteriocins use the Ton system (Ton system) to penetrate the outer membrane of sensitive bacteria, for example: colicin 5, 10, B, D, M, V, Ia, Ib… (Dimov et al., 2005; Kleanthous, 2010). In the general, group A colicins are encoded on small plasmids with a lysis gene and can be released out of the bacteria, while group B colicins are encoded on large plasmids without a lysis gene (Cascales et al., 2007).

**Table 1:** **Classification of colicins by different translocators system: Tol- and Ton-dependent in the *E. coli***.

**Colicins**	**Antibacterial activity**	**Receptor**	**Translocators**	**Molecular weight (Da)**	**Producing strain**	**References**
**GROUP A**						
A	Pore-forming	BtuB	OmpF, TolABQR	62989	*Citrobacter freudii*	Varenne et al., 1981; Morlon et al., 1983
E1	Pore-forming	BtuB	TolC, TolAQ	57279	*Escherichia coli*	Yamada et al., 1982
K	Pore-forming	Tsx	OmpAF, TolABQR	59611	*Escherichia coli*	Pilsl and Braun, 1995a,b,c
N	Pore-forming	OmpF	OmpF, TolAQR	41696	*Escherichia coli*	Pugsley, 1987
S4	Pore-forming	OmpW	OmpF, TolABQR	54085	*Escherichia coli*	Pilsl et al., 1999
U	Pore-forming	OmpA	OmpF, TolABQR	66289	*Shigella boydii*	Smajs et al., 1997
28b	Pore-forming	OmpA	OmpF, TolABQR	47505	*Serratia marcescens*	Guasch et al., 1995
						GenBank: CAA44310.1
E2	DNase	BtuB	OmpF, TolABQR	61561	*Escherichia coli*	Herschman and Helinski, 1967; Cursino et al., 2002
					*Shigella sonnei*	
E7	DNase	BtuB	OmpF, TolABQR	61349	*Escherichia coli*	Chak et al., 1991; Cursino et al., 2002
E8	DNase	BtuB	OmpF, TolABQR	~70000	*Escherichia coli*	Toba et al., 1988
E9	DNase	BtuB	OmpF, TolABQR	61587	*Escherichia coli*	Chak et al., 1991; Macdonald et al., 2004
E3	16S rRNase	BtuB	OmpF, TolABQR	57960	*Escherichia coli*	Herschman and Helinski, 1967; Cursino et al., 2002
E4	16S rRNase	BtuB	OmpF, TolABQR	ND	*Escherichia coli*	Males and Stocker, 1982
E6	16S rRNase	BtuB	OmpF, TolABQR	58011	*Escherichia coli*	Akutsu et al., 1989; Cursino et al., 2002
DF13	16S rRNase	IutA	OmpF, TolAQR	59293	*Escherichia coli*	van den Elzen et al., 1983
E5	tRNase	BtuB	OmpF, TolABQR	58254	*Escherichia coli*	Males and Stocker, 1982
					*Shigella sonnei*	GenBank: KF925332.1
**GROUP B**						
B	Pore-forming	FepA	TonB-ExbBD	54742	*Escherichia coli*	Schramm et al., 1987
Ia	Pore-forming	Cir	TonB-ExbBD	69429	*Escherichia coli*	Konisky and Richards, 1970 GenBank: AAA23182.2
Ib	Pore-forming	Cir	TonB-ExbBD	69923	*Escherichia coli*	Konisky and Richards, 1970
					*Shigella sonnei*	GenBank: AAA23188.1
5	Pore-forming	Tsx	TolC, TonB-ExbBD	53137	*Escherichia coli*	Pilsl and Braun, 1995a
10	Pore-forming	Tsx	TolC, TonB-ExbBD	53342	*Escherichia coli*	Pilsl and Braun, 1995b
D	tRNase	FepA	TonB-ExbBD	74683	*Escherichia coli*	Roos et al., 1989
M	Peptidoglycanase	FhuA	TonB-ExbBD	29453	*Escherichia coli*	Kock et al., 1987

*ND, not determined*.

When colicins enter the target cell, they can be divided into three categories based on bactericidal mechanisms: (1) Pore-forming type colicins: the formation of pores or channels in the inner-membrane cause leakage of cytoplasmic compounds, destruct electrochemical gradient, ion loss, and cell death. These include colicin A, B, E1, Ia, Ib, K, and N; (2) Nuclease type colicins: colicins containing DNase, 16S rRNase, and tRNase to non-specifically digest DNA and RNA of bacteria. These include colicin E2 to E9; (3) Peptidoglycanase type colicins: these proteins can digest the peptidoglycan precursor, leading to an inability to synthesize peptidoglycan and bacterial death (Cascales et al., 2007).

#### Microcins

Microcins are low molecular weight ribosomal synthesized hydrophobic antimicrobial peptides (<10 kDa), which is distinguished by 25–80 kDa high molecular weight colicins protein. Microcins are produced as precursor peptides, including N-terminal leader peptide and core peptides. Microcin precursor peptides may or may not undergo a post-translational modification process in the course of maturation to an active microcin. Microcins are predominantly produced by *Enterobacteriaceae* showing great tolerance to heat, extreme pH, and proteases (Rebuffat, 2012). The bactericidal mechanisms of microcins are diverse, including the pore-forming type, the nuclease type, such as DNase and RNase functions, and inhibitors of protein synthesis or DNA replication. No microcin genes have a corresponding lysis gene, and microcins are secreted outside the bacteria through the Type I ABC (ATP binding cassette) transporter secretion system, which is composed of a number of proteins (Duquesne et al., 2007).

Microcins are classified as two categories according to molecular masses, disulfide bonds in structure, and post-translational modifications (Table 2). Class I microcins, such as microcin B17, C7-C51, D93, and J25 are of low molecular weight (<5 kDa) post-translationally modified peptides. The molecular weight of class II microcins are larger (5–10 kDa) than that of class I microcins. Class II microcins can be further divided into two subclasses, including class IIa and IIb. Class IIa microcins, such as microcin L, V, and N require three different genes to synthesize and assemble functional peptides. Class IIb microcins, such as microcin E492, M, and H47, are linear peptides with or without post-translational modifications at the C-terminal (Severinov et al., 2007).

**Table 2:** **Classification scheme for gram-negative microcins**.

**Classification**	**Characteristics**	**Microcins**	**Molecular weight (Da)**	**Producing strain**	**References**
Class I	Low molecular weight peptides (<5 kDa), post-translationally modified	B17	3094	*Escherichia coli*	Collin et al., 2013
		C7/C51	1177	*Escherichia coli*	Severinov et al., 2007
		D93	<1000	*Escherichia coli*	Martinez and Perez-Diaz, 1986
		J25	2107	*Escherichia coli*	Wilson et al., 2003
Class II	Larger (5–10 kDa) peptides, with or without post-translational modifications				
class IIa	Required more than one genes to synthesize and assemble functional peptides	L	8884	*Escherichia coli*	Pons et al., 2004
		V	8741	*Escherichia coli*	Fath et al., 1994
		N/24	7274	*Escherichia coli*	Corsini et al., 2010
class IIb	Linear peptides with post-translational modifications or not at C-terminal	E492	7886	*Klebsiella pneumoniae*	Pons et al., 2002
		M	7284	*Escherichia coli*	Vassiliadis et al., 2010
		H47	4865	*Escherichia coli*	Vassiliadis et al., 2010

#### Bacteriocins from gram-positive bacteria

Unlike colicins from Gram-negative bacteria, which are plasmid or chromosome encoded 25–80 kDa proteins, the Gram-positive bacteria bacteriocins exert similar characteristics to microsins. These gene-encoded bacteriocins are low molecular weight antimicrobial peptides with less than 60 amino acids. In Gram-positive bacteria, lactic acid bacteria (LAB) are the typical bacteria producing a variety of bacteriocins of different sizes, structures, physicochemical properties, and inhibitory spectrum. Due to the large diversity of bacteriocins, some investigations show different ways to classify bacteriocins from Gram-positive bacteria (Dimov et al., 2005; Cotter et al., 2013). The Gram-positive bacteriocins are generally divided into class I (modified peptides, lantibiotics), class II (unmodified peptides, non-lanthionine), and class III (large proteins, heat unstable) (Table 3).

**Table 3:** **Classification scheme for gram-positive bacteriocins**.

**Classification/features**	**Bacteriocins**	**Molecular weight (Da)**	**Producing strain**	**References**
**CLASS I**				
The bacteriocins are post-translationally modified, linear or globular peptides containing lanthionine, β-methyl lanthionine and dehydrated amino acids	Nisin A	3352	*Lactococcus lactis subsp. lactic*	Field et al., 2012
	Nisin U	3029	*Streptococcus uberis*	Wirawan et al., 2006
	Nisin Z	3493	*Lactococcus lactis subsp. lactic*	Mulders et al., 1991
	Mersacidin	1824	*Bacillus sp.Y85,54728*	Chatterjee et al., 1992
	Labyrinthopeptin A2	1922	*Actinomadura sp*.	Meindl et al., 2010
	subtilosin A	3399	*Bacillus subtilis* 168	Babasaki et al., 1985
**CLASS II**				
Heat stable, unmodified, non-lanthionine-containing bacteriocins, heterogeneous class of small peptides				
Class IIa (pediocin PA-1like bacteriocins)	pediocin PA-1	4629	*Pediococcus acidilactici* PAC-1.0	Henderson et al., 1992
	carnobacteriocin X	3602	*Carnobacterium maltaromaticum* C2	Tulini et al., 2014
Class IIb (composed of two peptides)	lactacin F	4755	*Lactobacillus spp*.	Fremaux et al., 1993
	ABP-118	4096	*Lactobacillus salivarius subsp. salivarius* UCC118	Flynn et al., 2002
Class IIc (circular peptide)	carnocyclin A	5862	*Carnobacterium maltaromaticum* UAL307	Martin-Visscher et al., 2008
	enterocin AS-48	7149	*Enterococcus faecalis*	Samyn et al., 1994
Class IId (linear, non-pediocin like, single-peptide)	epidermicin NI01	6074	*Staphylococcus epidermidis*	Sandiford and Upton, 2012
	lactococcin A	5778	*Lactococcus lactis subsp. Cremoris*	Holo et al., 1991
**CLASS III**				
Large, heat unstable proteins	Caseicin 80	~42000	*Lactobacillus casei* B80	Muller and Radler, 1993
	Enterolisin A	34501	*Enterococcus faecalis* LMG 2333	Nilsen et al., 2003
	Helveticin J	37511	*Lactobacillus helveticus* 481	Joerger and Klaenhammer, 1990

Class I peptides are post-translationally modified bacteriocins or lantibiotics with less than 28 amino acids small membrane-active peptides (<5 kDa), linear or globular peptides which contain lanthionine, β-methyl lanthionine, and dehydrated amino acids. Linear structure peptides are membrane disrupting mode of action, and globular structure peptides with cellular enzymatic reaction. Class I bacteriocins are further subdivided into lantibiotics, such as linear peptide nisin and globular peptide mersacidin (Chatterjee et al., 1992), labyrinthopeptins, such as globular peptide labyrinthopeptin A2 (Meindl et al., 2010), and sactibiotics, such as globular peptide subtilosin A (Kawulka et al., 2004).

Class II bacteriocins are 30–60 amino acids (<10 kDa), which always exhibit the unique properties of heat tolerance, unmodified non-lanthionine, and positive charge. The class II bacteriocins are subdivided into five sub-classes by Cotter et al. (2013). Class IIa bacteriocins are *Listeria*-active peptides with a consensus amino acid sequence of YGNGVXaaC in the N-terminal, and include pediocin PA-1 (Henderson et al., 1992) and carnobacteriocin X (Tulini et al., 2014). Class IIb bacteriocins require two different unmodified peptides for forming a fully active poration complex, such as lactacin F (Allison et al., 1994) and ABP-118 (Flynn et al., 2002). Class IIc bacteriocins are circular peptide bacteriocins, such as carnocyclin A (Gong et al., 2009; Martin-Visscher et al., 2009), and enterocin AS-48 (Martínez-Bueno et al., 1994). Class IId bacteriocins are linear, non-pediocin-like, single-peptide bacteriocins, including epidermicin NI01 (Sandiford and Upton, 2012) and lactococcin A (Holo et al., 1991). Class IIe bacteriocins are non-ribosomal siderophore-type post-translation modification at the serine-rich carboxy-terminal region, such as microcin E492 (de Lorenzo and Pugsley, 1985). As microcin E492 was isolated from *Klebsiella pneumonia*, which is not a Gram-positive bacteria, the class IIe bacteriocins should be categorized to microcins of Gram-negative bacteria.

Class III bacteriocins are large molecular weight (>30 kDa), heat unstable proteins. Class III can be further subdivided into two distinct groups. Group A bacteriocins are the bacteriolytic enzymes which killing the sensitive strains by lysis of the cell well, such as Enterolisin A (Nilsen et al., 2003). Group B bacteriocins are non-lytic proteins such as Caseicin 80 (Müller and Radler, 1993) and Helveticin J (Joerger and Klaenhammer, 1986).

## Potential applications of bacteriocins in food science, pharmaceutics, and clinical medicine

**Figure 2:**
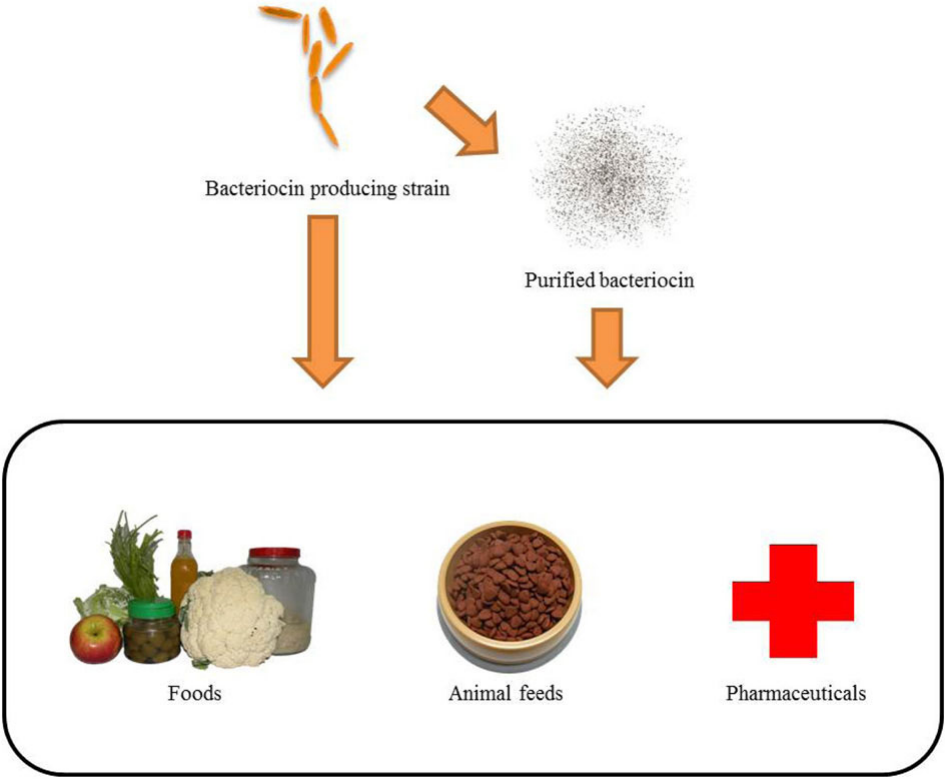
**Bacteriocin-producing strains and purified bacteriocins can be applied to food, animals, and medicine**. If bacteriocin-producing strains are applied through start culture or co-culture in food, it can extend the preservation of food. Probiotics produced by bacteriocins can balance the bacteria in the digestive tract to reduce gastrointestinal diseases. Purified bacteriocins can be added directly to foods as a natural preservative. Bacteriocins can be added to animal feed as an anti-pathogen additive to protect livestock against pathogen damage. The bacteriocins used in medicine can improve the quality of human life. Bacteriocins have the potential to replace antibiotics as an antibacterial drug, and are a novel anti-cancer drug.

Bacteriocins are now widely used in food science to extend food preservation duration (Ghrairi et al., 2012), which inhibit pathogen infection of animal diseases (van Heel et al., 2011), and pharmaceutical industry and medical society to treatment for malignant cancers (Figure 2) (Lancaster et al., 2007).

The first bacteriocin was discovered by Gratia (1925), and in recent years, many bacteriocins are successively identified by scientists. Bacteriocins are considered as a natural product because they are the peptides or proteins produced by bacteria present in many fermented or non-fermented foods since ancient times. Many microorganisms, such as bacteriocin producing LAB, are used to start cultures or co-cultures in food production processes for increasing flavor and prolonging shelf-life. In addition, many bacteriocin producing bacteria are isolated from foods and their raw materials (Settanni and Corsetti, 2008).

## Probiotics

The term “probiotic” is derived from the Greek *pro bios*, meaning “for life” or “in support of life,” which was first used by Lilly and Stillwell (1965). The probiotic is generally considered to promote the balance of intestinal microbiota and increase health benefits. The World Health Organization (WHO) defines probiotics as, “Live microorganisms which, when administrated in adequate amounts, confer a health benefit on the host” (Dobson et al., 2012). The characteristics of probiotics should include: a group of strains beneficial to the host animal that can stably survive and have metabolic activity in the intestinal environment, and being non-pathogenic and non-toxic, remain stable and viable for long periods of storage and harsh conditions (Fuller, 1989). There are many ways for probiotics to control intestinal pathogens. Probiotics demonstrate the capabilities of antimicrobial substance production, competitive exclusion of pathogen binding, competition for nutrients, and modulation of the immune system (FAO/WHO, 2001). Many antibacterial substances, such as bacteriocins, short chain fatty acids, and hydrogen peroxide, are produced by probiotics for inhibiting gastrointestinal microorganisms or pathogens. Dobson et al. (2012) considered that bacteriocins are one of the traits of probiotics. Currently many probiotics are used in daily life, including LAB, non-pathogenic *E. coli*, bacilli, and yeasts.

Purified bacteriocins, or bacteriocin producing probiotics, can reduce the number of pathogens or change the composition of intestinal microbiota in animal models, such as mice, chickens, and pigs. Bernbom et al. (2006) report the ability of pure nisin, nisin-producing *Lactococcus lactis* strain CHCC5826 and non-nisin-producing *Lactococcus lactis* strain CHCH2862, could affect the composition of intestinal microbiota in human flora-associated rats. They found that the number of *Bifidobacterium* in the feces of rats fed with nisin-producing and non-nisin-producing *Lactococcus lactis* for 8 days significantly increased, while the number of enterococci/streptococci in duodenum, ileum, cecum, and the colon were reduced. However, the above effect was not found after feeding of purified nisin. *Lactococcus lactis* may affect the intestinal microbiota through competition of nutrients or adhesion sites. Therefore, the effect of changes in the composition of intestinal microbiota may be not related to the presence of nisin in *Lactococcus lactis*. Cursino et al. (2006) indicated that colicin Ib, E1, and microcin C7, as derived from *E. coli* strain H22, possess the ability to inhibit the growth of pathogenic or non-pathogenic bacteria, including *Enterobacter, Escherichia, Klebsiella, Morganella, Salmonella, Shigella*, and *Yersinia*. In a germ-free mouse model, *E. coli* strain H22 showed the ability to reduce the population of *Shigella flexneri* 4 to undetectable levels in feces after a 6-day oral inoculation (Cursino et al., 2006). The results suggested that *E. coli* strain H22, and other multiple bacteriocin producing strains, had potential to be employed as a probiotic for livestock and humans. Corr et al. (2007) demonstrated that the human origin probiotic strains *Lactobacillus salivarius* UCC118 produced bacteriocin Abp118 for inhibiting infection with foodborne pathogen *Listeria monocytogenes* in mice. The bacteriocin Abp118 mutant strain UCC118Δ*abp118* failed to protect mice against infection by *Listeria monocytogenes*. Riboulet-Bisson et al. (2012) also demonstrated that bacteriocins of UCC118 were one of the points in changing the composition of microbiota in the mice and pig models. Millette et al. (2008) isolated two bacteriocin-producing LAB, *Lactococcus lactis* MM19 and *Pediococcus acidilactici* MM33, from human feces, and found the ability of LAB to reduce the numbers of vancomycin-resistant *Enterococci* (VRE) in the C57BL/6 mice model. Nisin Z and pediocin PA-1/AcH were produced by *Lactococcus lactis* MM19 and *Pediococcus acidilactici* MM33, respectively, which showed strong activity against clinical VRE isolate. The results revealed that *Lactococcus lactis* and non-pediocin PA-1/AcH producing mutant *Pediococcus acidilactici* MM33A had the ability to increase the total LAB and anaerobes populations, while *Pediococcus acidilactici* MM33 decreased the *Enterobacteriaceae* populations in healthy mice feces. Moreover, after feeding MM19 or MM33 for 3 days, the VRE populations in feces were respectively reduced to 2.50 and 1.85 log_10_ CFU/g than PBS control. A similar study (Dabour et al., 2009) demonstrated that pediocin PA-1 producing probiotic *Pediococcus acidilactici* UL5 had the ability to inhibit *Listeria monocytogenes in vitro*. However, *Pediococcus acidilactici* UL5 did not reduce the population of *Listeria monocytogenes* in the mouse intestinal, and was not detectable in fecal samples. *Pediococcus acidilactici* UL5 did not protect from infection of *Listeria monocytogenes*, which was due to the failure to compete with other intestinal microbes or inhibit the production of pediocin PA-1 by other microbial in the intestine. There is another ideal situation for artificially establishing probiotic, by transforming a bacteriocin gene into an *E. coli* strain. The benefits to humans by the more powerful probiotics can be produced using genetic engineering techniques. Gillor et al. (2009) utilized six different bacteriocin-encoding plasmids, including colicin A, E1, E2, E7, K, and N, in order to transform into the *E. coli* strain BZB1011. Four-week-old CD-1 mice were inoculated with the BZB1011 control strain, or one of the six colicinogenic BZB1011 strains. The fecal bacteria were monitored for 112 days. After 112 days incubation, the density of colicinogenicity BZB1011 in the feces was significantly higher than that of control BZB1011. The results suggest that colicin expression is helpful to increase *E. coli* colonization in the mouse gastrointestinal tract.

## Food technology

In order to extend shelf-life, antibiotics or food preservative are incorporated (e.g., nitrite and sulfur dioxide) into foods to delay microbial growth and possible corruption. However, most commercial preservatives are developed via chemical synthesis, and long-term consumption of the synthetic preservatives may have an adverse impact on the human body. Moreover, it is illegal to use antibiotics in food products. The bacteriocins produced by Gram-positive or Gram-negative bacteria are gene encoded peptides or proteins, which are suitable as natural preservatives in food products. Due to the sensitivity of bacteriocins to some proteases, harmless bacteriocins are possibly digested, thus, un-functional small peptides and amino acids are bacteriocin-loaded foods digested in the gastrointestinal tract (Cleveland et al., 2001; Bernbom et al., 2006). Thus, bacteriocins are considered as basically safe food additives after intake by the gastrointestinal system.

Bacteriocins are natural food additives due to the bacteriocin producing bacteria presence in many types of foods since ancient times, such as cheeses, yogurts, and Portuguese fermented meat (Yang et al., 2012; Todorov et al., 2014). In food technology, nisin is produced by *Lactococcus lactis* and was the first antibacterial peptide found in LAB (Rogers, 1928). It is also a commercial bacteriocin used as a food preservative against contamination by microorganism, which is marketed as Nisaplin®. It is the only bacteriocin approved for utilization as a preservative in many foods by the U.S. Food and Drug Administration (USFDA), and licensed as a food additive in over 45 countries (Settanni and Corsetti, 2008). Another commercially available bacteriocin is pediocin PA-1, marketed as Alta® 2341, which inhibits the growth of *Listeria monocytogenes* in meat products (Settanni and Corsetti, 2008). In many food products, such as traditional European cheeses, the milk used in the manufacturing process is easily contaminated with animal excrement. The bacteriocinogenic *Enterococci* as starter cultures or co-cultures can be used for reducing microbiota contamination (Foulquié Moreno et al., 2006). Settanni and Corsetti (2008) reviewed bacteriocinogenic LAB strains as a co-culture, protective, or starter cultures in fermented and non-fermented vegetables, such as olives, sourdough, miso, sauerkrauts, refrigerated pickles, and mungbean sprouts. Moreover, they introduced bacteriocins as foods additives, such as nisin, which is used in kimchi, mashed potatoes, and fresh-cut products. Enterocin AS-48 is used in cider, fruit and vegetable juices, and canned vegetables for contamination inhibition. Enterocin CCM4231 and EJ97 are used in soy milk and zucchini purée for suppression of contamination, respectively (Settanni and Corsetti, 2008).

## Treatment of pathogen-associated diseases

Since the first antibiotic penicillin was discovered in 1928 by Alexander Fleming, many discovered antibiotics are applied to treat pathogens. Antibiotics were first approved by USFDA in 1951, and used in animal feed, which significantly reduced the number of deaths due to bacterial infection cases. However, the problem with multiple drug resistance pathogens has become increasingly serious, due to concerns regarding the abuse of antibiotics (Joerger, 2003). Bacteriocins are reported to inhibit important animal and plant pathogens, such as Shiga toxin-producing *E. coli* (STEC), enterotoxigenic *E. coli* (ETEC), methicillin-resistant *Staphylococcus aureus* (MRSA), VRE, *Agrobacterium*, and *Brenneria* spp. (Grinter et al., 2012; Cotter et al., 2013). The bactericidal mechanism of bacteriocins are mainly located in the receptor-binding of bacteria surfaces, and then through the membrane, which causes bacteria cytotoxicity. In addition, bacteriocins are low-toxic peptides or proteins sensitive to proteases, such as trypsin and pepsin (Cleveland et al., 2001).

Jordi et al. (2001) found that 20 kinds of *E. coli* could express colicin, which inhibited five kinds of Shiga toxin-producing *E. coli* (O26, O111, O128, O145, and O157: H7). These *E. coli* can cause diarrhea and hemolytic uremic syndrome in humans. In a simulated cattle rumen environment, colicin E1, E4, E8-J, K, and S4 producing *E. coli* can significantly inhibit the growth of STEC. Stahl et al. (2004) utilized purified colicin E1 and colicin N for effective activity against enterotoxigenic *E. coli* pathogens F4 (K88) and F18 *in vitro*, which caused post-weaning diarrhea in piglets. Furthermore, purified coilicin E1 proteins were mixed with the dietary intake of young pigs. The results showed a reduction of the incidence of post-weaning diarrhea by F-18 positive *E. coli* (Cutler et al., 2007). The growth of the piglets was thus ameliorated. Józefiak et al. (2013) used the nisin-supplemented bird diet to feed broiler chickens, and found a reduced number of *Bacteroides* and *Enterobacteriacae* in ileal digesta of nisin-supplemented chickens. The action of nisin was similar to that of salinomycin. After a 35-day growth, the average body weight gain of nisin-supplemented (2700 IU nisin/g) chickens is 1918 g/bird, which was higher than the 1729 g of non-nisin supplemented or the 1763 g of salinomycin-supplemented chickens. Stern et al. (2006) reported that class II low molecule mass bacteriocin OR-7 was purified from the *Lactobacillus salivarius* strain NRRL B-30514. This bacteriocin exhibited an ability against human gastroenteritis pathogen *Campylobacter jejuni*. OR-7 was stable when treated with lysozyme, lipase, and heat to 90°C, or at pH ranges from 3.0 to 9.1. The purified OR-7 was encapsulated in polyvinylpyrrolidone for chicken feed. The populations of *C. jejuni* were reduced at least one million fold over that of non-OR-7 supplemented chickens in cecal material of OR-7-treated chickens. These results suggest that nisin, OR-7, and other bacteriocins, illustrated potential when applied to replace antibiotics in poultry and other animal feeds.

## Cancer therapy

Over the past half century, cancer has become a serious problem, and a threat to human health. According to new information from the WHO website, there were 8.2 million people died from cancer and 14.1 million new cancer cases worldwide in 2012, with 60% of world's total new annual cases occurring in Africa, Asia, and Central and South America. Of the world's top 10 leading causes of death, lung cancer (2.7%, including trachea and bronchus cancer) was 7th and caused 1.5 million (2.7%) deaths in 2011, higher than 1.2 million (2.2%, 9th) deaths in 2000. In the United States, there were 1,660,290 new cancer cases, with 580,350 cancer deaths projected to occur in 2013, pedestalling cancer as the second leading cause of death, exceeded only by heart diseases (Center, 2013; Siegel et al., 2013).

In cancer therapy, some researches indicate that bacteriocins show activity against tumor cells. Considering that bacteriocins are naturally and legally added in foods, bacteriocins may be suitable as a potential anti-tumor drug candidate. Some bacteriocins, such as pore-forming colicin A and E1 inhibited the growth of one human standard fibroblast line MRC5 and 11 human tumor-cell lines (Chumchalová and Smarda, 2003). Contrarily, pore-forming colicin U and RNAase activity colicin E3 did not display this growth inhibition capability. Colicin D, E2, E3, and pore-forming colicin A could inhibit the viability of murine leukaemia cells P388, while pore-forming colicin E1 and colicin E3 suppressed v-myb-transformed chicken monoblasts (Fuska et al., 1979; Lancaster et al., 2007). Bures et al. (1986) isolated *E. coli* from the feces of 77 colorectal carcinoma patients, where 32 patients (41.6%) had barteriocins-producing *E. coli*. In the feces of 160 healthy people, 102 people (63.8%) had barteriocins-producing *E. coli*, which also showed that colicins from bacteria in the intestine may be one of the factors in reducing human colorectal carcinoma. Colicins could act as an anti-cancer drug of moderate potential. Supplements of bacteriocin-producing probiotics may be another way to prevent cancer occurrence. In a recent study, Joo et al. (2012) found that nisin had capabilities to prevent cancer cell growth. Three head and neck squamous cell carcinoma (HNSCC) 17B, HSC, and 14A were treated by nisin at concentrations of 40 and 80 μg/ml. After 24 h nisin increased DNA fragmentation or apoptosis, arrested cell cycle and reduced cell proliferation of HNSCC occurred. The floor-of-mouth oral cancer xenograft mouse model was used to test the anti- HNSCC function of nisin. A 150-mg/kg dose of nisin was administered orally every day for 3 weeks, and tumor volumes were significantly reduced in nisin-treated mice, as compared with the control mice, which received only water. These results indicated that nisin provides a safe and novel therapy for treating HNSCC.

## Conclusions and future perspectives

In general, the genes and immune genes of bacteriocins are encoded on the same plasmid or in adjacent regions of a chromosome. The bactericins genes can enter into other bacterial cells via conjugation (Ito et al., 2009; Burton et al., 2013). Accordingly, by conjugation or transposon for insertion, the bacterial pathogen can obtain some immune genes of bacteriocins. In this way, in environment or food pathogens prevention strategies, the use of purified bacterial peptide or protein is superior to the use of bacteria to produce bacteriocins. In terms of disease control, many papers report only the ability to fight pathogens of the probiotic produced by a single or specific bacteriocin, and the ability of the probiotic generated by a single bacteriocin to balance the bacteria strains in the intestinal tract. However, there are many different kinds of pathogens and mutants in nature. The specific use of a particular bacteriocin cannot eliminate all bacterial pathogens. Therefore, we can mix a variety of bacteriocin proteins to make cocktail drugs for application to the prevention of certain human or animal pathogens to cause death or reduce its diffusion rate. In disease control, bacteriocin can solve some of the most challenging problems of multi-drug resistant pathogens.

In recent years, the increased number of multi-drug resistant pathogens has become a serious problem, and finding or developing a new generation of antimicrobial agents is becoming increasingly important. Many new antibacterial substances have also been found by scientists to replace the old antibiotics; however, finding and identifying new antimicrobial substances is a difficult task, and the use of biotechnology to fuse two known bacteriocins into a new bacteriocins may be a quick fix method. By using recombinant PCR techniques, Acuña et al. (2012) integrated enterocin CRL35 and microcin V genes to obtain a bacteriocins called Ent35-MccV. Ent35-MccV has bactericidal capacity against clinically isolated enterohemorrhagic *E. coli* and *Listeria monocytogenes*, as well as some Gram-positive and Gram-negative bacteria, which are not clinically isolated. Such a technique can crease new or multi-functional bacteriocins, which are more powerful in functionality and germicidal range. As a result, they can be widely used in food, animal husbandry, and medicine.

### Conflict of interest statement

The authors declare that the research was conducted in the absence of any commercial or financial relationships that could be construed as a potential conflict of interest.
