# Corrigendum: Antibacterial activities of bacteriocins: application in foods and pharmaceuticals

**DOI:** 10.3389/fmicb.2014.00683

**Published:** 2014-12-05

**Authors:** Shih-Chun Yang, Chih-Hung Lin, Calvin T. Sung, Jia-You Fang

**Affiliations:** ^1^Research Center for Industry of Human Ecology, Chang Gung University of Science and TechnologyTaoyuan, Taiwan; ^2^Pharmaceutics Laboratory, Graduate Institute of Natural Products, Chang Gung UniversityTaoyuan, Taiwan; ^3^Chang Gung Memorial HospitalTaoyuan, Taiwan; ^4^Center for General Education, Chang Gung University of Science and TechnologyTaoyuan, Taiwan; ^5^Chronic Diseases and Health Promotion Research Center, Chang Gung University of Science and TechnologyTaoyuan, Taiwan; ^6^Department of Microbiology, Immunology, and Molecular Genetics, University of California, Los AngelesLos Angeles, CA, USA; ^7^Chinese Herbal Medicine Research Team, Healthy Aging Research Center, Chang Gung UniversityTaoyuan, Taiwan

**Keywords:** bacteriocin, protein, natural product, food, cancer treatment

An additional affiliation of “Chang Gung Memorial Hospital, Kweishan, Taoyuan, Taiwan” was attached for Shih-Chun Yang.

## Conflict of interest statement

The authors declare that the research was conducted in the absence of any commercial or financial relationships that could be construed as a potential conflict of interest.

